#  Role of Fructose as a Potent Antiarrhythmic and Anti-infarct agent in Isolated Rat Heart 

**Published:** 2014

**Authors:** Mahsa Haghighat Azari, Moslem Najafi

**Affiliations:** a Student Research Committee, Faculty of Pharmacy, Tabriz University of Medical Sciences, Tabriz, Iran.; b Department of Pharmacology and Toxicology, Faculty of Pharmacy, Tabriz University of Medical Sciences, Tabriz, Iran.; c Biotechnology Research Center, Tabriz University of Medical Sciences, Tabriz, Iran.

**Keywords:** Fructose, Ischemia/reperfusion, Arrhythmia, Infarction, Isolated rat heart

## Abstract

In the current study, effects of acute short term administration of fructose on cardiac arrhythmias and myocardial infarction size following ischemia/reperfusion were investigated in isolated rat heart. The hearts were subjected to 30 min zero flow global ischemia followed by 120 min reperfusion. In the control group, the hearts were perfused by normal drug free Krebs-Henseleit (K/H) solution throughout the experiments, while in the treated groups (2-4), they were perfused with fructose containing K/H solution at 12, 24 and 48 mM concentrations during stabilization and reperfusion time, respectively. Cardiac arrhythmias were determined based on the Lambeth conventions and the infarct size was measured by computerized planimetry. Myocardial infarction size was 22 ± 3% in the control group, however administration of fructose (12, 24 and 48 mM) reduced it to 15 ± 3 (P<0.05), 7±2 (P<0.001) and 4 ± 2% (P<0.001), respectively. A direct linear correlation between fructose concentrations and infarction size reduction was observed (R^2^=0.970). In addition, total number of ventricular ectopic beats were significantly decreased by all used concentrations of fructose (P<0.01 for group 2, P<0.001 for groups 3 and 4, respectively). Fructose also produced significant decrease in the number, incidence and duration of ventricular tachycardia compared to the control (P<0.05). The data showed that acute short term administration of fructose can protect isolated rat heart against ischemia/reperfusion injuries as reduction of infarct size and cardiac arrhythmias. Alterations in glycogen storage and/or glycolytic efficiency may probably involve in these cardioprotective effects. Also it is possible that fructose can act as a pharmacological preconditioning agent.

## Introduction

Myocardial infarction is one of the most important causes of mortality and morbidity in the world (1). It is defined as necrosis of heart muscle caused by ischemia (2) and is part of the spectrum of acute coronary syndromes, which includes unstable angina, non-Q wave, and Q wave myocardial infarction (3). More than 20 years, coronary reperfusion therapy has been used to manage myocardial infarction. However, reperfusion following prolonged ischemia itself can lead to ischemia/reperfusion (I/R) injury (1). Clinical manifestations of this injury include angina, myocardial necrosis, arrhythmia, myocardial stunning and endothelial dysfunction (4). Although the heart may adapt chronically to ischemia by increasing of blood flow through collateral vessels, clinical interventions such as thrombolysis, percutaneous coronary angioplasty or coronary bypass surgery eventually are needed to overcome the underlying ischemia (4). 

Many environmental factors are involved in the development of cardiovascular diseases. There is a direct relationship between these factors and human diets. In recent years, there has been a tremendous increase in the consumption of carbohydrates in human diets (5, 6). The most common sugars in our nutrition are sucrose, fructose, and glucose. Fructose (fruit sugar) is an important part of the daily energy intake (7, 8). It is a simple monosaccharide with a chemical formula C_6_H_12_O_6_ found in many natural sources (9).

For many past centuries humans consumed fructose amounting to 16-20 g per day, largely from fresh fruits. Today, a significant increase in fructose daily consumptions amounting to 85-100 g is a routine. This large quantities of fructose consumptions leads to lipogenesis and triglyceride accumulation, which in turn results in insulin sensitivity reduction and hepatic insulin resistance/glucose intolerance. It has been found that fructose intakes above 25% of total energy consumed will cause hypertriglyceridemia and gastrointestinal symptoms (10). These adverse effects of fructose are the reason that its metabolism has gained recent research attention (10). Interestingly, small quantities of fructose improve glucose tolerance and decrease the glycemic response to glucose loads. Some previous studies have shown that fructose-fed rats are protected against I/R injury (11, 12). On the other hand, results of some other investigations demonstrated that fructose had a destructive effect on ischemic reperfused heart (13). Most of the previous studies in this field were focused mainly on the long term impact of fructose on the ischemic heart, however, in the present study, effects of acute short term administration of fructose on I/R-induced injuries (cardiac arrhythmias and myocardial infarction size) were investigated during global ischemia followed by reperfusion in isolated rat heart.

## Experimental

*Chemicals*

The following chemicals were purchased: Triphenyltetrazolium chloride (TTC) (Sigma), Formalin, NaCl, NaHCO_3_, KCl, KH_2_PO_4_, MgSO_4_, CaCl_2_, D-glucose, D-fructose (Merck, Germany), Sodium pentobarbital (Kela Company, Belgium) and Heparin (Daru-Pakhsh Company, Iran). 

*Animals and surgical procedure*

Male Wistar rats (weighing 270-330 g) were used in this study. The rats were divided into four groups randomly as a control and three treated groups (n=7-10 in each group). The animals were pretreated with intraperitoneal (*i.p*) injection of 300 IU heparin then anaesthetized by sodium pentobarbital (50-60 mg/Kg, *i.p*). After that, their hearts were excised rapidly and mounted on a non-recirculating langendorff apparatus under constant pressure of 100 mmHg at 37 °C. Finally, the hearts were perfused with modified Krebs-Henseleit (K/H) solution which was freshly prepared and equilibrated with 95% O_2_-5% CO_2_. A latex fluid filled balloon was inserted into the left ventricle and inflated to give a preload of 8-10 mmHg (14). All the hearts were subjected to 30 min stabilization, 30 min zero flow global ischemia followed by 120 min reperfusion. In the control group, the hearts were perfused by normal K/H solution throughout the experiments, while in the treatment groups (2, 3 and 4), they were perfused with normal K/H solution plus 12, 24 and 48 mM fructose during stabilization time and 120 min reperfusion, respectively. An epicardial ECG was recorded continuously by a physiograph during the experiment. Based on the Lambeth conventions (15), the ECGs were analyzed to determine the number of single ectopic beats, salvos (couplets and triplets), ventricular tachycardia (VT), the total number of ventricular ectopic beats (VEBs), incidence and duration of VT and reversible ventricular fibrillation (Rev VF), incidence of irreversible ventricular fibrillation (Irrev VF) and total VF during the first 30 min of reperfusion time (16). 

*Measurement of myocardial infarction size*

To determine the infarction size, at the end of 120 min reperfusion, the hearts were frozen, and then sliced transversely in a plane perpendicular to the apico-basal axis into 2 mm thick sections. The slices were incubated by 1% (w/v) TTC solution in phosphate buffer (pH=7.4) for 15 min at 37 °C to dye the non-infarcted region (17). This procedure resulted in the non-infarcted, non-perfused tissue stained brick red and infarcted tissue remaining unstained and appeared pale. After fixing the slices in formalin 10% (v/v) for 24 h, the infarct size was determined by using a computerized planimetty package (18, 19). The experiments reported were carried out in accordance with the Guide for the Care and Use of Laboratory Animals (National Institutes of Health Publication No 85-23, revised 1985).

*Statistical analysis*

All the results are expressed as Mean ± SEM except for the incidence of VT and VF which are expressed as percentage. To compare the number of VEBs, single ectopic beats, salvos, VT and duration of VT and VF between groups, the Mann-Whitney non-parametric U-test was employed. Fisher exact test (Chi-square with Yates correction) was used to analyze the incidence of VT and VF. The mean percentage of infarct size was analyzed using one-way ANOVA and then significant differences were examined by LSD post hoc range test. Differences between groups were considered significant at a level of P<0.05.

## Results

**Figure 1:**
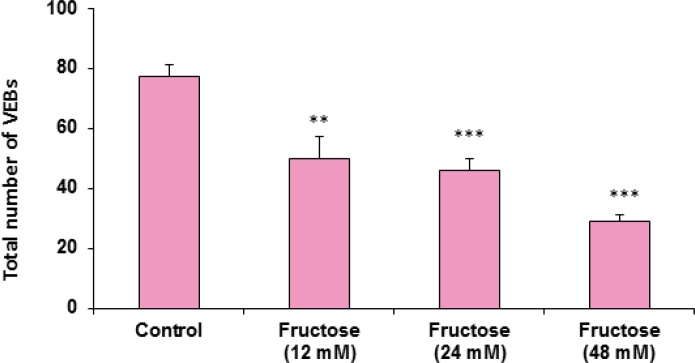
The total number of ventricular ectopic beats (VEBs: Single+Salvos+VT) in the control and treated groups receiving 12, 24 and 48 mM of fructose in K/H solution (groups 2, 3 and 4). Data are represented as Mean ± SEM. **P<0.01, ***P<0.001 *versus* the control group. N=7-10 in each group

**Figure 2:**
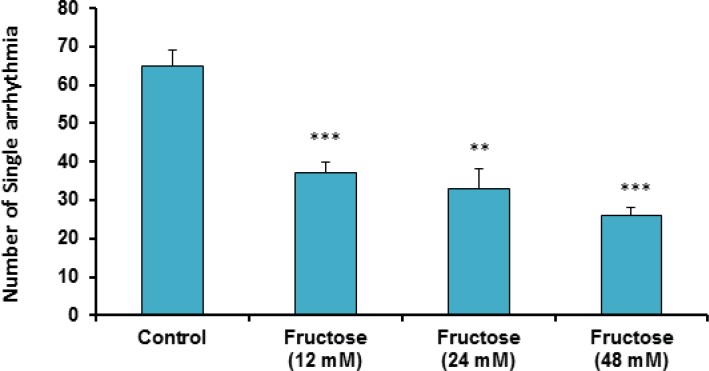
Number of single arrhythmias in the control and treated groups receiving 12, 24 and 48 mM of fructose in K/H solution. Data are represented as Mean ± SEM. **P<0.01, ***P<0.001 *versus* the control group. N=7-10 in each group

The effects of acute high concentrations of fructose against reperfusion-induced cardiac arrhythmias after 30 min zero flow global ischemia are summarized in Table 1. As shown in the table and Figure 1, perfusion of K/H solution containing fructose in groups 2, 3 and 4 significantly decreased the total number of VEBs. In the control group, number of VEBs was 77 ± 4, while the value was decreased by administration of fructose (12, 24 and 48 mM) to 50 ± 7 (P<0.01), 46 ± 4 (P<0.001) and 29 ± 2 (P<0.001), respectively. As illustrated in Figure 2, using fructose also markedly lowered the number of single ectopic beats compared to the control group (P<0.01 for group 3, P<0.001 for groups 2 and 4, respectively). Likewise, fructose at 24 and 48 mM concentrations produced significant decrease (P<0.05) in the number, incidence and duration of VT compared to the control value (Table 1). At the same time, it had no significant reduction in the number of salvos arrhythmias. The time spent in reversible VF was 27 ± 14 sec in the control group, however administration of fructose reduced VF duration not statistically significant. In addition, fructose containing K/H solution lowered the incidence of total VF by 12, 24 and 48 mM, although the effect was not significant compared to the control group (Table 1).

**Table 1:** Effects of fructose administration (12, 24 and 48 mM) on reperfusion-induced cardiac arrhythmias after 30 min zero flow global ischemia in isolated rat hearts.

**Groups**	**Reperfusion-induced cardiac arrhythmias**						
	**VEBs** **number**	**VT** **number**	**VT duration** **(sec)**	**Rev VF** **duration** **(sec)**	**Rev VF** **incidence** **(%)**	**Irrev VF Incidence** **(%)**	**VT** **incidence** **(%)**
**Control**	4±77	2±8	1±4	14±27	30	0	50
**Fructose** **(12 mM)**	**7±50	1±5	1±2	10±15	20	0	20
**Fructose** **(24 mM)**	***4±46	* 0	* 0	4±10	14	0	* 0
**Fructose** **(48 mM)**	***2±29	* 0	* 0	5±7	14	0	* 0

* P<0.05,

** P<0.01 and

*** P<0.001* versus *the control group. N=7-10 in each group. VT: Ventricular Tachycardia, VEBs: Ventricular Ectopic Beats (Single+Salvos+VT), Rev VF: Reversible Ventricular Fibrillation, Irrev VF: Irreversible Ventricular Fibrillation.

**Figure 3:**
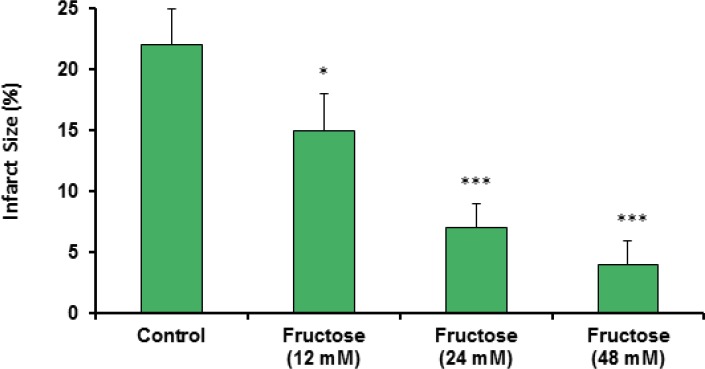
Myocardial infarction size in the control and treated groups receiving 12, 24 and 48 mM of fructose in K/H solution. Data are represented as Mean±SEM. *P<0.05, ***P<0.001 *versus* the control group. N=7-10 in each group

**Figure 4:**
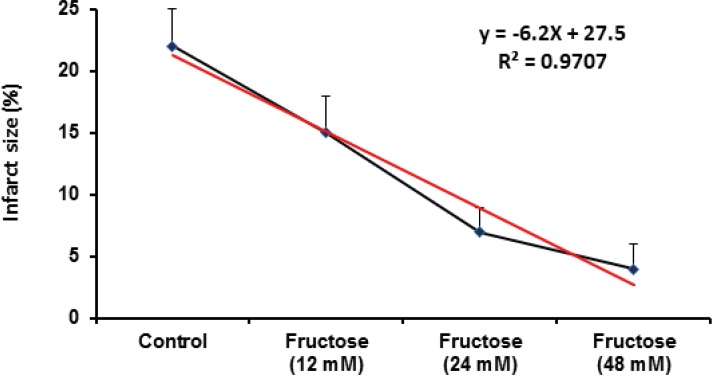
Relationship between myocardial infarct size reduction and fructose concentrations (0-48 mM) in isolated rat hearts. Data are represented as Mean±SEM

**Figure 5:**
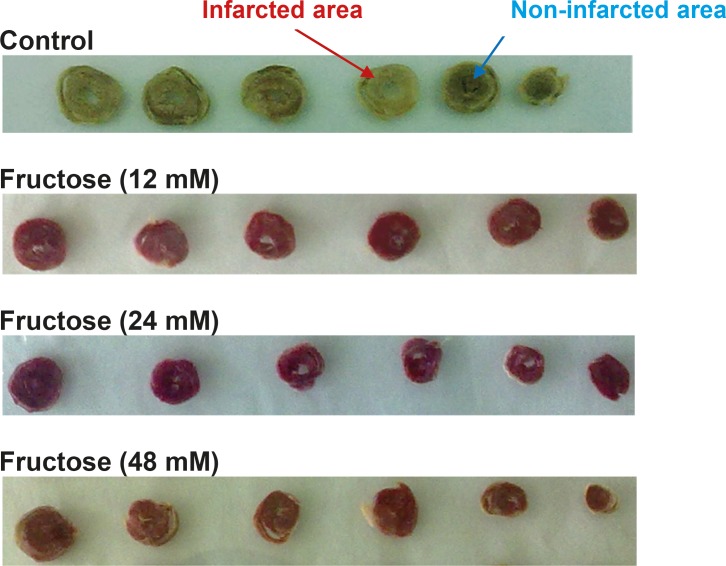
Representative heart sections from the control and treated groups receiving 12, 24 and 48 mM of fructose stained by triphenyltetrazolium chloride. The infarcted (dead) and non-infarcted (alive) areas are seen white (pale) and brick red, respectively.

Effects of fructose on the myocardial infarction size are summarized in Table 2. Perfusion of the isolated rat hearts by K/H solution containing fructose markedly reduced infarct size by the all used concentrations. As shown in Figure 3, the infarct size was 22 ± 3% in the control group, while fructose (12, 24 and 48 mM) produced significant reduction in the size of myocardial infarction from the control value to 15 ± 3% (P<0.05), 7 ± 2% (P<0.001) and 4 ± 2% (P<0.001), respectively. In addition, administration of fructose significantly decreased the infarcted volume of ischemic hearts in comparison with the control group (Table 2). Furthermore, a direct linear correlation (R^2^=0.970) between fructose concentrations and reduction of infarct size was observed (Figure 4). Sample staining images to determine infarction size by TTC method in isolated rat heart have been illustrated in Figure 5.

**Table 2:** Effects of fructose administration (12, 24 and 48 mM) on myocardial infarction size after 30 min zero flow global ischemia followed by 120 min reperfusion in isolated rat hearts

**Infarct size (%)**	**Infarcted vol. (mm** ^3^ **)**	**Groups**
3±22	41±209	**Control**
*3±15	**16±119	**Fructose** **(12 mM)**
***2±7	***15±62	**Fructose** **(24 mM)**
***2±4	***5±19	**Fructose** **(48 mM)**

Data are represented as Mean±SEM.

* P<0.05,

** P<0.01 and

*** P<0.001* versus* the control group. N=7-10 in each group.

## Discussion

Cardiac arrhythmias are major cause of mortality and morbidity throughout the world especially in developed countries (20). In the course of cardiac surgery and myocardial infarction, ventricular arrhythmias such as VT and VF are the most important cause of mortality (21). The results of this work showed that perfusion of fructose containing K/H solution can protect isolated rat hearts against reperfusion-induced cardiac arrhythmias after 30 min zero flow global ischemia. In this condition, different concentrations of fructose significantly reduced the total number of VEBs and the number of single arrhythmias compared to the control group. In addition, number, incidence and duration of VT were reduced. We also observed that incidence and duration of Rev VF and incidence of total VF were decreased non-significantly in the treated groups.

Some previous studies have shown that administration of high fructose diets (typically 50-60% of total energy intake) is a common way to induce features of the metabolic syndrome in rodents (22). Fructose intake causes endothelial dysfunction within 2 weeks (23) with activation of both the sympathetic nervous system (24) and rennin-angiotensin system (25) and stimulation of oxidative stress (26). This is associated with a rise in blood pressure, the development of insulin resistance and hypertriglyceridemia (27). Fructose can also cause weight gain and increased abdominal fat in rodents (28). Some studies suggest that weight gain is independent of total energy intake, suggesting an effect on basal metabolic rate. One possible mechanism is that fructose may cause leptin resistance (22). Maybe, the potential combination of hypertension and obesity with severe metabolic changes will stimulate cardiac dysfunction. For example, in metabolic syndrome, the heart has an increased mass, altered diastolic function, and the patients are prone to heart failure (29). In addition, fructose feeding during chronic pressure overloads induced hypertrophy and contractile dysfunction in the heart. In such condition, chronic pressure overload switches myocardial oxidative energy metabolism from fatty acids to glucose and impairs mitochondrial ATP production and the transfer of ATP to the contractile element (30). However, most studies deal with high or very high fructose concentrations, which are not relevant to daily fructose consumption in humans (31). The results of a previous study in 2006 revealed that chronic administration of fructose (feeding of rats by a diet in which 58% of the total carbohydrate was fructose for 4 weeks) had a protective effect against I/R-induced injury. The authors hypothesized that the increase in plasma vitamin E level induced by 4 weeks of fructose feeding prevents oxidative stress during I/R, thus mediating the protection afforded by this diet in the isolated rat hearts (12). Likewise, Jordan et al. observed the same cardioprotection in rats fed a high-fructose diet for only 3 days and showing normal fasting glucose and insulin levels. They concluded that fructose feeding induces cardioprotection via a preconditioning phenomenon (11).

In spite of some methodological differences between the present and the above studies (such as type of ischemia, experimental protocols and the administration period of fructose), findings of this work are in consistent with the above investigations. That is, acute, chronic and short time administrations of fructose protect isolated rat heart against I/R-induced injuries. As proposed by Jordan et al., cardioprotective effect of fructose could be specific to fructose itself and not secondary to the metabolic abnormalities associated with it. The mechanism by which fructose feeding is protective during myocardial I/R is not known. However, we theorize that alterations in glycogen storage and/or glycolytic efficiency may be responsible. Indeed, fructose bypasses the main regulatory step of glycolysis (the conversion of glucose-6-phosphate to fructose 1,6-bisphosphate) and as a result it can continuously enter the glycolytic pathway (6). During hypoxia and ischemia, myocardial energy production switches from the use of fatty acids to carbohydrates, thereby allowing maintenance of adequate ATP synthesis when oxygen availability is limited (11). There is huge data indicating that increased preischemic glycogen stores may provide protection against myocardial ischemic insult (32, 33). Moreover, it has been shown that small amounts of dietary fructose increase glycogen storage in the liver (34, 35). Therefore, it is possible that administration of large amounts of fructose may increase myocardial glycogen stores (11). Additionally, previous *in-vitro* studies in liver have shown fructose to be protective during both hypoxia and anoxia (-). The mechanism for this protection is purportedly due to an increased production in ATP during anaerobic metabolism (11). Thus, it is possible that fructose increases glycogen storage that in turn provides energy to the myocardium during ischemia (11). Obviously, this mechanistic proposal is speculative; however, it is partially clear from the current data that administration of fructose containing K/H solution to the ischemic heart alters the myocardial metabolism in some way such that it is protected from ischemia (11). 

Our findings also demonstrated that acute short term administration of fructose caused significant and potent cardioprotection against myocardial infarction as one of the most important determinants of I/R-induced injuries (Figure 3 and Table 2). Regarding the used concentration range of fructose in our model, reduction of infarct size is concentration dependent and there is a direct linear relationship (with an equation of y=-6.2x+27.5, r^2^=0.9707) between fructose concentration and its protective effect. Therefore, the higher concentrations of fructose are more effective than lower concentrations in decreasing myocardial infarction. Clinically, the current data provide the idea that short term pretreatment with high concentration of fructose could be a useful option for protecting the heart from cardiac surgery-induced I/R injuries. Considering the results of some previous experimental studies, it seems that fructose can up-regulate its own pathways. After the absorption in the gastrointestinal tract, fructose is transported to the liver then enters hepatocytes via the glucose transporter Glut5-independently of insulin (39). Incubation of liver cells, adipocytes or kidney tubular cells with fructose results in increasing fructokinase levels and of their respective transporters (Glut2 and Glut5) (22). In addition, feeding fructose to rats increases Glut5 expression in the intestines (40) and fructokinase activity in liver and intestines (41, 42). It is possible that these mechanisms are present in cardiomyocytes although further experiments are required to elucidate these mechanisms.

## Conclusion

The results of current study showed that acute short term administration of fructose can protect isolated rat hearts and consequently has antiarrhythmic activity and reducing infarction size. The mechanism for this protection is probably due to an increased production in ATP during anaerobic metabolism at ischemic conditions. Thus, it is possible that fructose increases glycogen storage that in turn provides energy to the myocardium during ischemia so that protects the heart against reperfusion injuries. Furthermore, in regard to using fructose before ischemia, it is possible that this agent can act as a pharmacological preconditioning factor. Future studies are required to determine the exact protective mechanisms of fructose.
